# Diffuse Large B-Cell Lymphoma Presenting Primarily as a Cutaneous Leg Lesion: A Case Report and Literature Review

**DOI:** 10.7759/cureus.75735

**Published:** 2024-12-15

**Authors:** Ahmed Hebishy, Aakriti Arora, Mohamed Maher, Srijan Valasapalli, Darla Liles

**Affiliations:** 1 Internal Medicine, East Carolina University (ECU) Health Medical Center/Brody School of Medicine, Greenville, USA; 2 Hematology and Medical Oncology, East Carolina University (ECU) Health Medical Center/Brody School of Medicine, Greenville, USA; 3 Pathology and Laboratory Medicine, East Carolina University (ECU) Health Medical Center/Brody School of Medicine, Greenville, USA

**Keywords:** chemotherapy, diffuse large b-cell lymphoma (dlbcl), immunohistochemistry (ihc), primary cutaneous diffuse large b-cell lymphoma - leg type, r-mini-chop

## Abstract

Primary cutaneous B-cell lymphoma (PCBCL) has three subtypes, among those, the leg type variant is the rarest with the highest rates of relapse and recurrence making it an intriguing focus for researchers. Nevertheless, prior to framing a diagnosis solely based on the lesion's location, it is prudent to reconsider whether it is genuinely a primary cutaneous B-cell lymphoma (PCBCL) or if it aligns more closely with the more prevalent lymphoma variants such as diffuse large B-cell lymphoma (DLBCL) with cutaneous involvement.

We are reporting a case of an 85-year-old African American lady, who presented with unilateral left leg DLBCL with cutaneous involvement. The patient was treated for recurrent cellulitis that was non-responsive to antibiotics; this prompted evaluation by leg MRI, which revealed a mass-like lesion involving the skin and muscle. Additional workup, which included tissue diagnosis and immunohistochemistry (IHC) studies, confirmed the diagnosis of DLBCL. A thorough clinical, laboratory, and imaging workup revealed diffuse meningeal lymphomatous involvement on brain MRI. The patient was started on reduced-intensity rituximab, cyclophosphamide, doxorubicin, vincristine, and prednisone (R-mini-CHOP) protocol with granulocyte-colony stimulating factor (G-CSF) and showed a good initial response. This case highlights the importance of comprehensive evaluation in distinguishing primary cutaneous B-cell lymphomas from other variants, particularly in atypical presentations such as diffuse involvement.

## Introduction

Diffuse large B-cell lymphoma (DLBCL) is the most common histologic subtype of non-Hodgkin lymphoma (NHL). It accounts for approximately 25% of NHL cases in the developed world [1]. The incidence of DLBCL in the United States is approximately seven cases per 100,000 persons/year [2].

In around 40% of cases, the disease arises in extranodal, extramedullary tissues [3]. The most common site of primary extranodal disease is the stomach/gastrointestinal tract, with cutaneous lymphoma ranking second in frequency. Primary cutaneous large B-cell lymphoma, leg type (PCLBCL-LT), is a relatively new subtype of B-cell lymphomas that could be easily framed when extranodal lymphomas present primarily on the legs. We present an interesting case of DLBCL that presented initially solely with leg lesion, creating an initial misimpression suggestive of PCLBCL-LT.

## Case presentation

An 85-year-old African American lady presented with a three-month history of gradually growing painful lesion on her left leg. Her past medical history includes hypertension and diverticulitis with enterocolic fistula that required sigmoid colectomy and colostomy.

**Figure 1:**
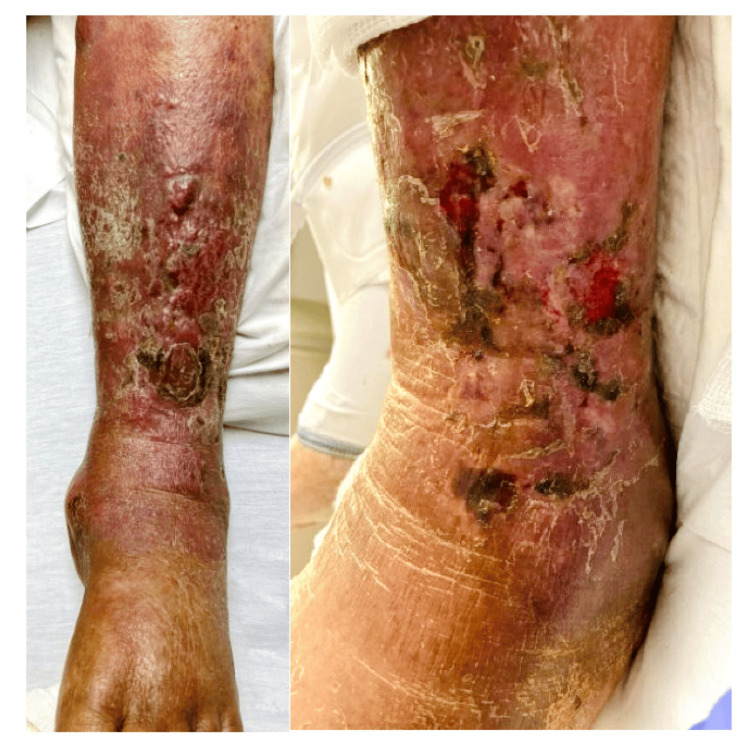
Skin lesion on presentation.

The lesion primarily affected the anterior aspect of the lower half of her left leg. It appeared as reddish-purple, nodules mainly over the shaft of the left tibia, surrounded by edematous, scaly skin with crusts (Figure 1). For a suspected diagnosis of cellulitis, she received three consecutive courses of different antibiotics (cephalexin, Augmentin, and levofloxacin) without much improvement, but rather progression. Lab work was remarkable for pancytopenia on complete blood count (CBC). Initial labs are shown in Table 1.

**Table 1:** Initial basic laboratory results.

Parameter	Results	Normal range
White blood cells (WBC) (x10³/µL)	3.05	4.5-11.0
Neutrophil (%)	66	75
Red blood cells (RBC) (x10⁶/µL)	2.32	4.5-5.9
Hemoglobin (g/dL)	7.4	12-16
Platelets (x10³/µL)	108	150-450
Sodium (Na) (mmol/L)	142	135-145
Potassium (K) (mmol/L)	3.6	3.5-5.0
Blood urea nitrogen (BUN) (mg/dL)	18	7-20
Creatinine (mg/dL)	0.78	0.6-1.3
Calcium (Ca) (mg/dL)	8.3	8.5-10.5
Magnesium (Mg) (mg/dL)	1.8	1.7-2.2
Uric acid (mg/dL)	3.5	3.5-7.2 (males), 2.6-6.0 (females)
Aspartate aminotransferase (AST) (U/L)	16	10-40
Alanine aminotransferase (ALT) (U/L)	19	7-56
Thyroid-stimulating hormone (TSH) (mIU/L)	2.31	0.35-4.94

**Figure 2:**
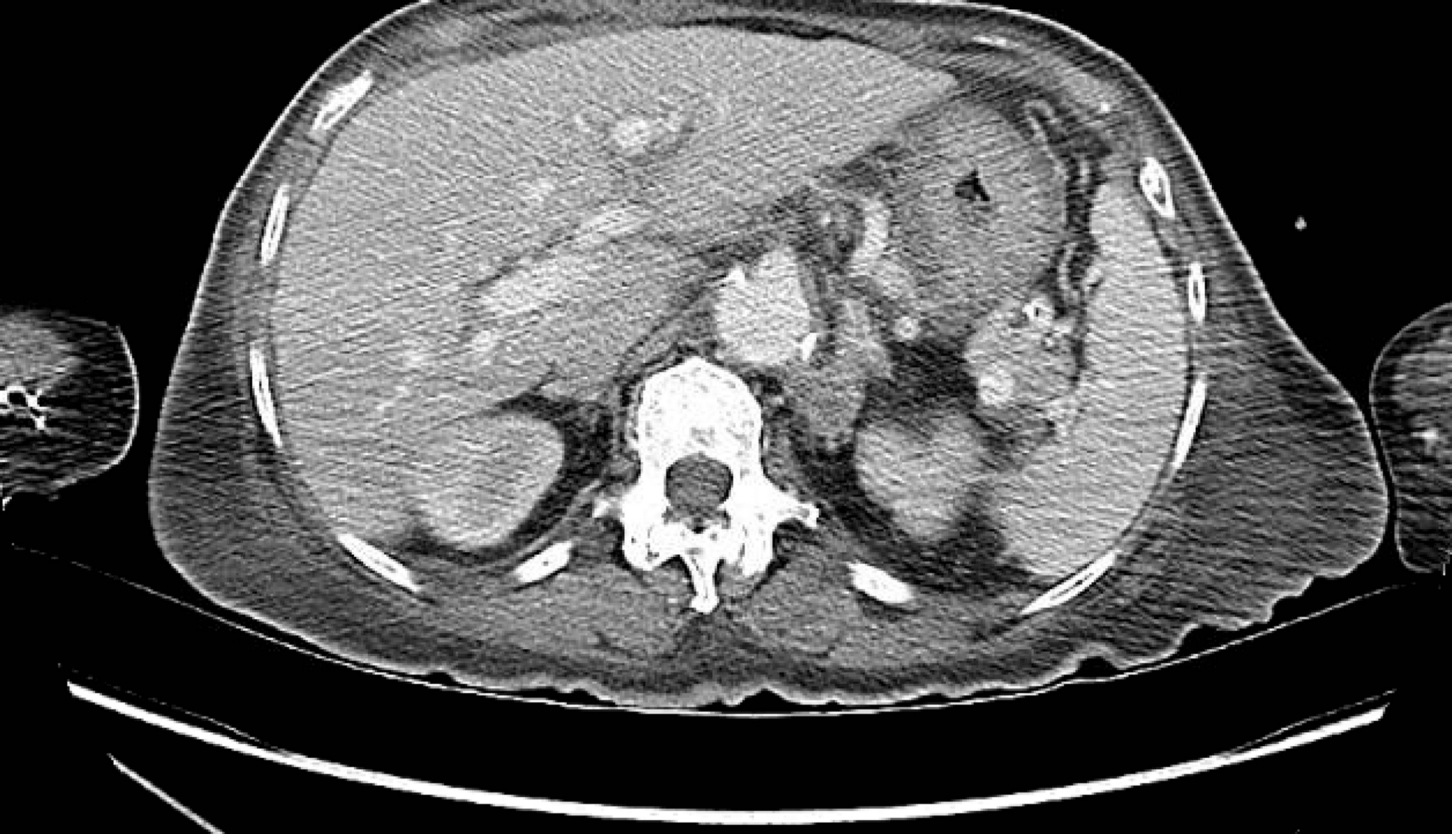
CT abdomen and pelvis. The image shows left retroperitoneal, periaortic lymphadenopathy.

**Figure 3:**
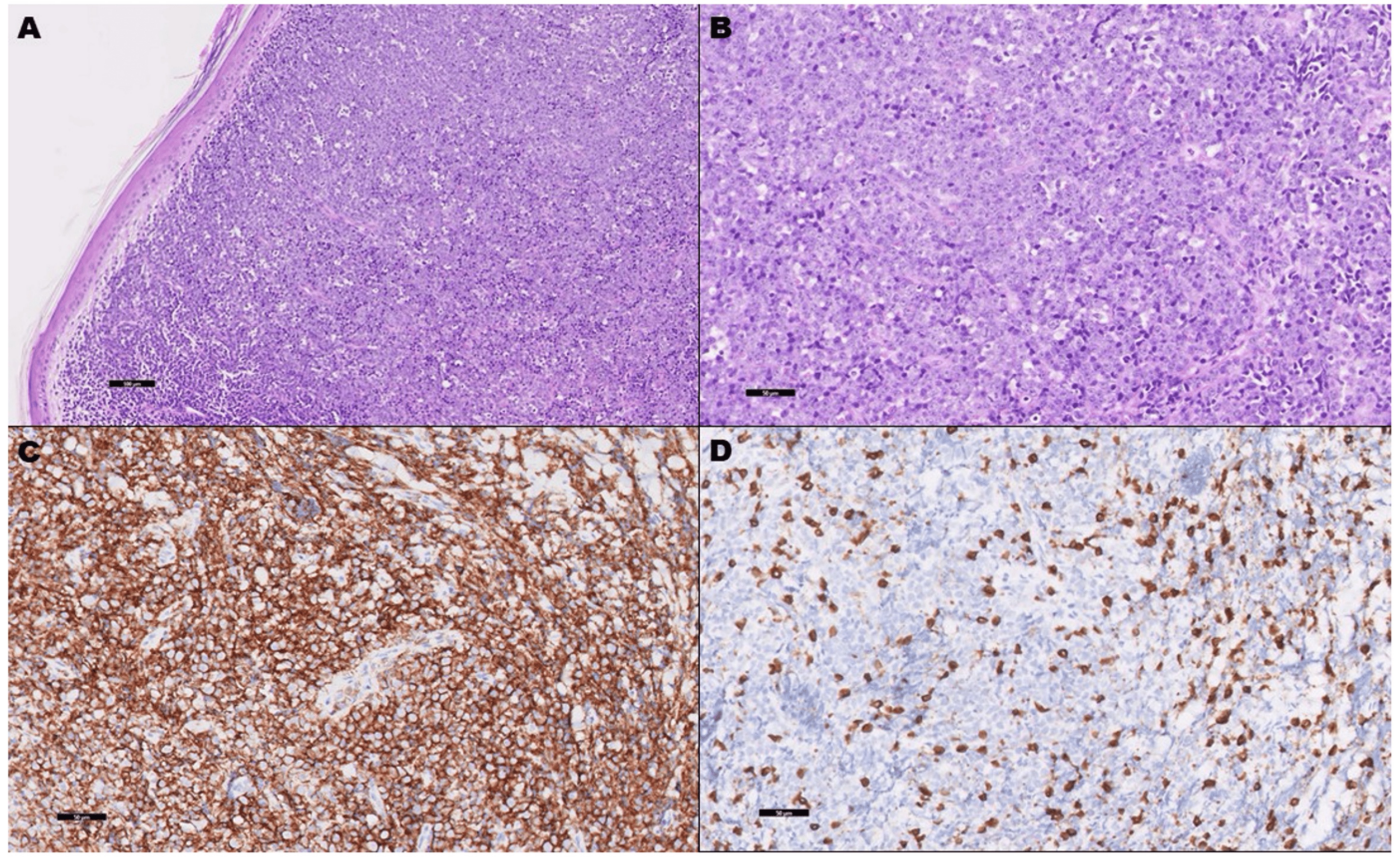
Skin biopsy histopathologic evaluation. The images show (A) hematoxylin and eosin-stained sections demonstrating diffuse atypical lymphoid infiltrate effacing the dermis (100x); (B) higher magnification shows medium-to-large sized cells, with crowding, prominent nucleoli, and high mitotic count (200x); and the atypical cells are diffusely positive for CD20 (C) while negative CD3 (D), highlighting only background reactive T-cells (200x).

**Figure 4:**
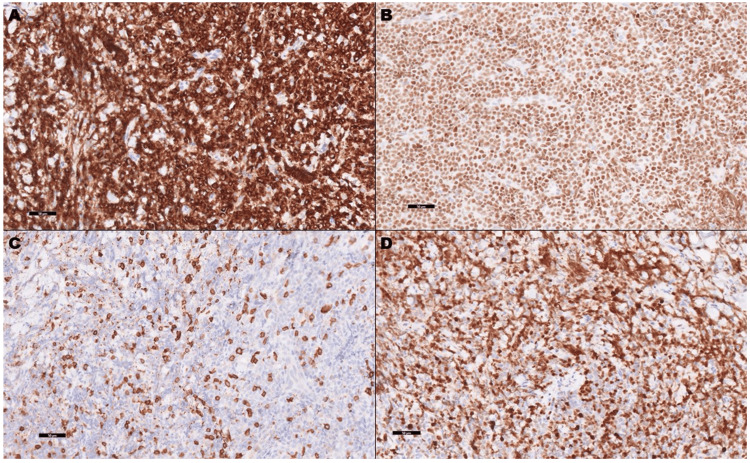
Immunohistochemical analysis showing marker expression. Additional immunohistochemical evaluation of the atypical cells showed diffuse immunoreactivity for BCL-2 (A) and C-Myc (B). Scattered atypical cells were positive for CD5 (C). Ki-67 proliferation index (D) was increased by approximately 60-70% (200x). BCL-2: B-cell lymphoma 2; C-Myc: cellular myelocytomatosis proto-oncogene

With consideration of neoplastic etiology, an MRI of the left leg was performed revealing focal regions of mass-like subcutaneous induration along the medial aspect of the proximal left lower leg. Similar lesions within the muscle body along the lateral margin of the mid-fibular shaft were also noted. CT abdomen and pelvis revealed a left retroperitoneal and periaortic lymphadenopathy (Figure 2). Skin biopsy and histopathologic evaluation of the left ankle biopsy revealed lymphoma (Figures 3A-3D) with Ki67 demonstrating an increased proliferation rate of approximately 60-70% (Figures 4A-4D).

**Figure 5:**
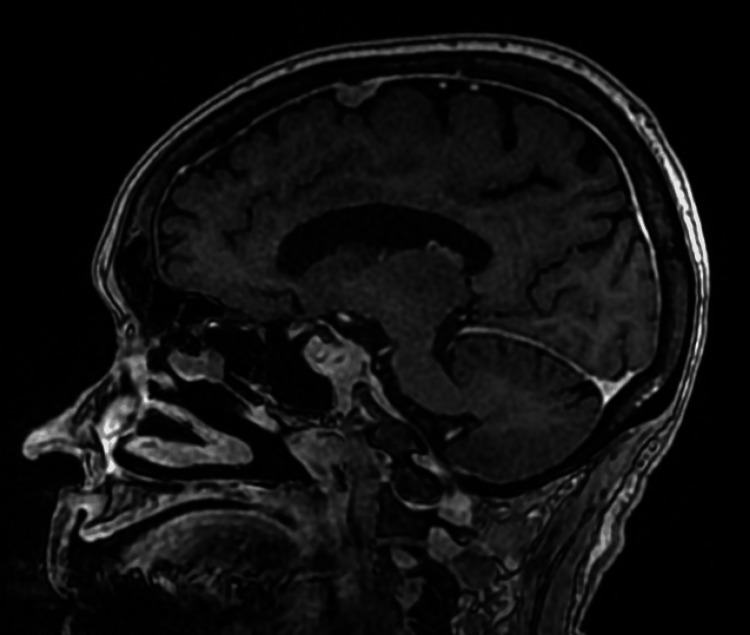
Post-contrast brain MRI showing diffuse smooth dural enhancement consistent with lymphomatous infiltration.

The differential diagnosis mainly included cutaneous involvement by systemic diffuse large B-cell lymphoma versus primary cutaneous diffuse large B-cell lymphoma, leg type. That distinction might be challenging solely on the histologic evaluation. However, dim positivity for CD5 was seen within a few of the large cell infiltrates. This expression is rarely reported in primarily cutaneous DLBCL, leg type. Therefore, based on the radiologic findings, combined with the morphologic and immunophenotypic characteristics, the diagnosis was more consistent with a diffuse large B-cell lymphoma, activated B-cell type, with cutaneous involvement. In the setting of encephalopathy, MRI brain was performed, and it showed diffuse smooth dural enhancement with lymphomatous infiltration (Figure 5). A subsequent lumbar puncture was performed, and she received prophylactic intrathecal methotrexate injection. CSF didn’t yield malignant cells.

She was eventually found to have central nervous system (CNS) involvement. The patient was commenced on rituximab, cyclophosphamide 400 mg/m^2^, vincristine 1 mg, doxorubicin 25 mg/m^2^, and prednisone 40 mg/m^2^ (R-mini-CHOP protocol) with concurrent granulocyte-colony stimulating factor (G-CSF). Our discussion explores the management of this case in light of existing published data.

## Discussion

Most diffuse large B-cell lymphomas originate in lymph nodes but ≤40% initially present in extranodal sites, with cutaneous lymphomas representing the second most frequent extranodal lymphoma, after the gastrointestinal tract [4]. A study by Takahashi et al. found that skin involvement in DLBCL was associated with a significant decrease in the overall survival rate [5]. Sixty percent of DLBCL patients will present with advanced stage (usually stage III or IV disease), while 40% have a more localized disease [6].

The patient in this study presented with a cutaneous lesion located on the left leg. Depending on the site of involvement, the following two distinct subsets of lymphoma may be implicated: PCDLBCL-LT versus extranodal cutaneous DLBCL. Distinguishing between these two types is crucial for accurate patient prognosis, as they each have distinct treatment approaches and survival outcomes.

Although skin lesion characteristics do not differ significantly in both settings, extensive cutaneous lesions are more often observed in extranodal cutaneous DLBCL compared with PCDLBCL-LT. Primary cutaneous B-cell lymphoma (PCBCL) refers to cases that are only confined to the skin with no evidence of extracutaneous disease at the time of diagnosis and after the completion of an initial staging evaluation [7].

Microscopically, the morphology of skin lesions can be similar among both entities, and immunohistochemistry (IHC) by itself is typically insufficient to distinguish between primary and systemic DLBCL. Since PCDLBCL-LT most commonly involves the lower leg(s) of older women, this initially created a premature misimpression in our case. Further evaluation revealed disease extension into the leg muscles beneath the primary lesion, a suspicious metastasis to a left retroperitoneal periaortic lymph node, and central nervous system involvement confirmed by brain MRI. Cells were diffusely positive for CD45, PAX-5, and CD20 on IHC stains and CD5 demonstrated dim positivity in the large cell infiltrate, making the diagnosis of PCLBCL-LT very unlikely. The diagnosis was more consistent with a diffuse large B-cell lymphoma, activated B-cell type.

Diffuse large B-cell lymphoma (DLBCL) typically exhibits rapid growth. The standard treatment often involves chemotherapy, commonly using a combination of four drugs known as cyclophosphamide, doxorubicin, vincristine, and prednisone (CHOP) regime, along with the monoclonal antibody, rituximab [8].

Treatment with six cycles of R-CHOP was associated with 90% six-year overall survival (OS) and 80% progression-free survival (PFS) in patients aged 18-60 years in the MInT trial [9]. R-mini-CHOP achieved a 75% two-year progression-free survival (PFS) and an 81% two-year OS in patients over 21 years old [10]. In patients aged >80 years with sufficient cardiac, renal, and hepatic function, as well as those aged 60-80 years with modest impairments, R-mini-CHOP is favored to minimize adverse effects that are typically associated with more aggressive regimens [11]. Our patient did well on R-mini-CHOP and was discharged to inpatient rehabilitation for further functional management.

## Conclusions

In conclusion, this study emphasizes the importance of heightened awareness regarding the cutaneous presentation of diffuse large B-cell lymphomas, particularly in the absence of distinctive clinical features that differentiate them from primary cutaneous lymphomas, leg type. The lack of clear-cut characteristics underscores the challenges in accurate diagnosis and underscores the critical need for increased vigilance among clinicians. Recognizing and understanding these subtle nuances is essential for ensuring appropriate management and optimizing outcomes for individuals affected by lymphomas of cutaneous presentation. Multidisciplinary approach is essential for navigating the complexities of skin lesions associated with lymphoma and ensuring optimal patient care.
